# Crystal structures of *catena*-poly[[μ-aqua-di­aqua(μ_3_-2-methyl­propano­ato-κ^4^ *O*:*O*,*O*′:*O*′)calcium] 2-methyl­propano­ate dihydrate], *catena*-poly[[μ-aqua-di­aqua­(μ_3_-2-methyl­propano­ato-κ^4^ *O*:*O*,*O*′:*O*′)strontium] 2-methyl­propano­ate dihydrate] and *catena*-poly[[μ-aqua-di­aqua­(μ_3_-2-methyl­propano­ato-κ^4^ *O*:*O*,*O*′:*O*′)(calcium/strontium)] 2-methyl­propano­ate dihydrate]

**DOI:** 10.1107/S2056989020012888

**Published:** 2020-09-25

**Authors:** Erika Samolová, Jan Fábry

**Affiliations:** a Inst. of Physics of the Czech Academy of Sciences, Na Slovance 2, 182 21 Praha 8, Czech Republic

**Keywords:** layered crystal structure, hydrogen bonding, carboxyl­ates, the Cambridge Structural Database, solid solution

## Abstract

The crystal structures of *catena*-poly[[μ-aqua-di­aqua­(μ_3_-2-methyl­propano­ato-κ^4^ *O*:*O*,*O*′:*O*′)calcium] 2-methyl­propano­ate dihydrate], {[Ca(C_4_H_7_O_2_)(H_2_O)_3_](C_4_H_7_O_2_)·2H_2_O}_*n*_, (I), *catena*-poly[[μ-aqua-di­aqua­(μ_3_-2-methyl­propano­ato-κ^4^ *O*:*O*,*O*′:*O*′)strontium] 2-methyl­propano­ate dihydrate], {[Sr(C_4_H_7_O_2_)(H_2_O)_3_](C_4_H_7_O_2_)·2H_2_O}_*n*_, (II), and *catena*-poly[[μ-aqua-di­aqua­(μ_3_-2-methyl­propano­ato-κ^4^ *O*:*O*,*O*′:*O*′)(calcium/strontium)] 2-methyl­propano­ate dihydrate], {[(Ca,Sr)(C_4_H_7_O_2_)(H_2_O)_3_](C_4_H_7_O_2_)·2H_2_O}_*n*_, (III), are related. (III) can be considered as an Sr-containing solid solution of (I), with Ca^2+^ and Sr^2+^ occupationally disordered in the ratio 0.7936 (16):0.2064 (16). (I)/(III) and (II) are homeotypic with different space groups of *Pbca* and *Cmce*, respectively. All the title crystal structures are composed of hydro­philic sheets containing the cations, carboxyl­ate groups as well as water mol­ecules. The hydro­phobic layers, which consist of 2-methyl­propano­ate chains, surround the hydro­philic sheets from both sides, thus forming a sandwich-like structure extending parallel to (001). The cohesion forces within these sheets are the cation–oxygen bonds and O—H⋯O hydrogen bonds of moderate strength. Stacking of these sandwiches along [001] is consolidated by van der Waals forces. The structures contain columns defined by the cation–oxygen inter­actions in which just one symmetry-independent 2-methyl­propano­ate anion is included, together with three water mol­ecules. These mol­ecules participate in an irregular coordination polyhedron composed of eight O atoms around the cation. Additional water mol­ecules as well as the second 2-methyl­propano­ate anion are not part of the coordination sphere. These mol­ecules are connected to the above-mentioned columns by O—H⋯O hydrogen bonds of moderate strength. In (II), the Sr^2+^ cation, two of the coordinating water mol­ecules and both anions are situated on a mirror plane with a concomitant positional disorder of the 2-methyl­propyl groups; the non-coordinating water mol­ecule also shows positional disorder of its hydrogen atom.

## Abstract 2

The crystal structures of [Ca(C_4_H_7_O_2_)(H_2_O)_3_](C_4_H_7_O_2_)·2H_2_O, space group *Pbca*, (I), and [Sr(C_4_H_7_O_2_)(H_2_O)_3_](C_4_H_7_O_2_)·2H_2_O space group *Cmce* (II) are homeotypic; [(Ca,Sr)(C_4_H_7_O_2_)(H_2_O)_3_](C_4_H_7_O_2_)·2H_2_O, space group *Pbca*, (III), is an Sr-containing solid solution of (I) with Ca^2+^ and Sr^2+^ occupationally disordered in the ratio 0.794 (2):0.206 (2).

## Chemical context   

A search of the Cambridge Structural Database (Groom *et al.*, 2016 ▸; version 5.41 with updates until August 2020) for crystal structures containing solely alkaline earth cations and 2-methyl­propano­ate (or isobutyrate) anions revealed hexa­kis­[bis­(μ_2_-2-methyl­propano­ato)(2-methyl­propanoic acid)mag­nes­ium], refcode NAGQUI (Coker *et al.*, 2004 ▸) and *catena*-poly[[tri­aqua­(isobutyrato-*kO*)magnesium]-μ-isobutyrato-*κ* ^2^ *O:O′*] monohydrate, refcode VIQTOG (Malaestean *et al.*, 2013 ▸). Although limited to these two examples, some basic structural features of these compounds can be inferred from other simple carboxyl­ate salts. These features, among others, are illustrated by the series of structures determined by Coker *et al.* (2004 ▸), in which the number of carbon atoms in the carboxyl­ate anions gradually increases.
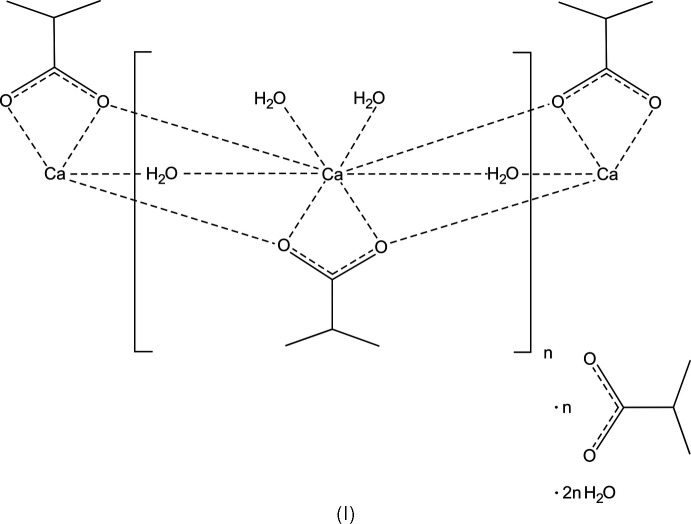
 
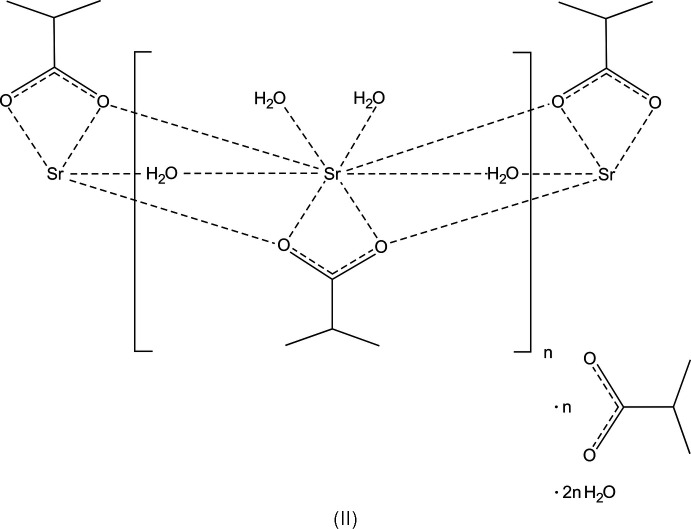
 
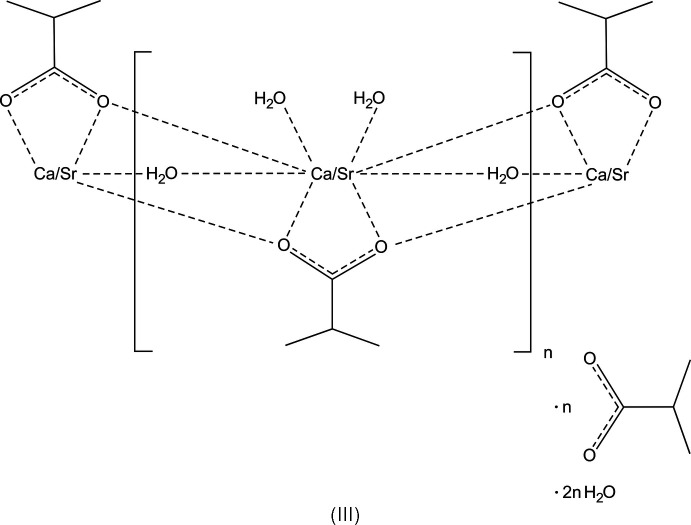


In the crystal structure of *catena*-[tetra­kis­(μ_2_-formato)tetra­aqua­dimagnesium], MGFORD03 (Coker *et al.*, 2004 ▸), no hydro­phobic organic chain is present. In the other member of this series, bis­(μ_2_-acetato-*O*,*O*,*O*′)-tetra­kis­(μ_2_-acetato-*O*,*O*′)bis­(acetic acid)di­aqua­trimagnesium acetic acid solvate, NAGQOC [Coker *et al.* (2004 ▸), see also the redetermination of this structure by Scheurell *et al.* (2012 ▸), NAGQOC02], there are sheets within the structure separated into hydro­philic parts (composed of the cations and oxygen atoms) and hydro­phobic parts (composed of methyl groups). The remaining free acetic acid mol­ecules are bound by O_acetic_—H⋯O hydrogen bonds between the layers. NAGQUI is an example of a structure where the hydro­philic part is surrounded by a hydro­phobic layer. The same holds for hexa­kis­[bis­(μ_2_-3,3-di­methyl­butanato)(3,3-di­methyl­butanoic acid)magnesium], NAGRET (Coker *et al.*, 2004 ▸), as well as for bis­(pivalato)tetra­kis­(pivalic acid)magnesium, VAMCUI01 [Coker *et al.* (2004 ▸), see also VAMCUI determined by Troyanov *et al.* (2002 ▸)]. Thus, the longer the organic chain, the more important the van der Waals forces become for mol­ecular cohesion in structures with carboxyl­ate anions. The different cohesion forces in the hydro­philic and the hydro­phobic parts are the reason for the formation of layer-like structures or structures where an organic part completely surrounds a hydro­philic metal–oxygen sheet or a hydro­philic cluster. Likewise, the longer the hydro­phobic chains, the larger is the probability of inclusion of non-coordinating water mol­ecules into the structure because the latter can provide binding bridges between the carboxyl­ate anions, which would otherwise be isolated. Such a situation is realised in VIQTOG where the water mol­ecules complete a column substructure that is defined by the cation–oxygen bonds stemming from the carboxyl­ate groups and water mol­ecules. The growing complexity of water substructures with a growing number of carbon atoms in carboxyl­ate anions has also been observed in the salts of the first five di­carb­oxy­lic acids with 4,6-di­amino­pyrimidine (Matulková *et al.*, 2017 ▸).

The present study was undertaken to prepare dicalcium strontium hexa­kis­(2-methyl­propano­ate) with the intention that the resulting crystal structure might be related to dicalcium strontium hexa­kis­(propionate) **(**CASRPP06; Mishima, 1984 ▸), which exhibits inter­esting structural and physical properties (*e.g.* Itoh, 1992 ▸). However, the synthesis attempt resulted in one of the title structures, *catena*-poly[[μ-aqua-di­aqua­(μ_3_-2-methyl­propano­ato-κ^4^ *O*:*O*,*O*′:*O*′)(calcium/stront­ium)] 2-methyl­propano­ate dihydrate], (III). We then also prepared the pure Ca and Sr compounds, *i.e.* (I) and (II), the crystal structures of which are also reported here.

## Structural commentary   

The structures have the same features and are composed of the respective cation, two carboxyl­ate mol­ecules and additional water mol­ecules. One of the carboxyl­ate anions and three water mol­ecules coordinate to the cation, the remaining mol­ecules form a substructure inter­connected by hydrogen bonds only. Compound (III) is an Sr-containing solid solution of (I), and the two structures are crystal-chemically isotypic. The refined ratio of the occupationally disordered cation site is Ca:Sr = 0.7936 (16):0.2064 (16). The crystal structures of (I)/(III) and (II) are homeotypic (Lima-de-Faria *et al.*, 1990 ▸), with similar lattice parameters and crystal-chemical features, but different space-group types.

**Figure 1:**
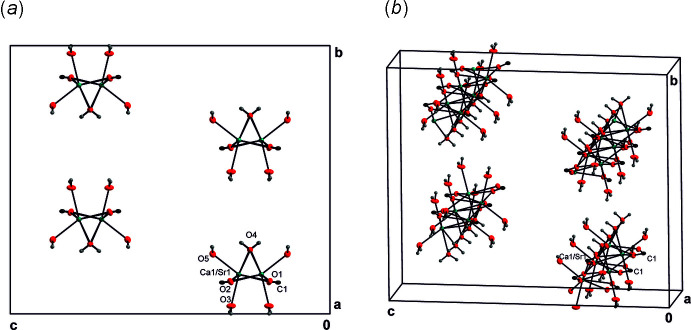
(*a*) View of the columns along the *a* axis in the crystal structure of (III). The columns depicted are formed by (Ca1/Sr1) (green) and O atoms (red); the latter are also depicted with bonds to carbon C atoms (grey) and H atoms (light-grey spheres of arbitrary radius). Displacement ellipsoids are shown at the 30% probability level. (*b*) Perspective view of the columns in (III).

There are three main cohesion forces present in the title structures: The first cohesion force regards the cation–oxygen inter­actions. For each of the crystal structures, there are eight oxygen atoms in the coordination sphere, defined by one carboxyl­ate mol­ecule in a bidentate bridging mode. (In VIQTOG there are two carboxyl­ate anions coordinating in a monodentate mode and bridging to other Mg^2+^ cations.) In the title structures, the cation-coordinating atoms are symmetry-equivalent atoms O1 in (II), and O1 and O2 in (I) and (III), respectively. Other coordinating O atoms are the water O atoms O2, O3 and O4 in (II), and the water O atoms O3, O4 and O5 in (I) and (III). [The Sr^2+^ cation in (II) is located on a mirror plane (Wyckoff position 8*f*)]. Numerical values of the cation–oxygen bonds are listed in Tables 1 ▸, 2 ▸ and 3 ▸ for structures (I), (II) and (III), respectively. The coordination polyhedra form columns oriented parallel to the *a* axis. Because of the similarity of the three structures, (III) was chosen as a representative (Fig. 1 ▸ *a*,*b*).

**Figure 2:**
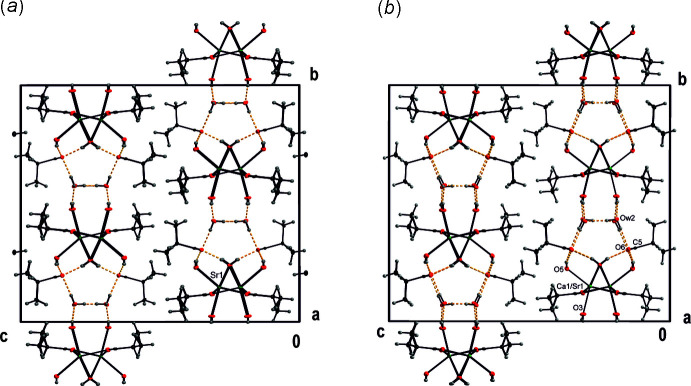
View of the unit-cell content of (*a*) (II) and (*b*) (III). Hydrogen bonds are shown as yellow dashed lines; colour code as in Fig. 1 ▸. The substructures with the hydro­philic sheets and hydrogen-bonded system, which connects the columns and water mol­ecules, are clearly discernible from the hydro­phobic part of the structure composed of 2-methyl­ethyl chains.

**Figure 3:**
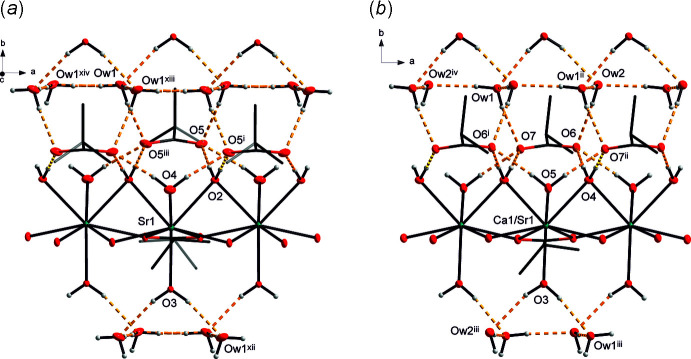
View of the hydrogen-bonded substructures in (*a*) (II) and (*b*) (III). The symmetry codes correspond to those given in Tables 5 ▸ and 6 ▸, respectively. Colour code is as in Fig. 1 ▸.

The second type of a cohesion force in the title structures originates from O—H⋯O hydrogen bonds of moderate strength (Gilli & Gilli, 2009 ▸) that link the above mentioned columns into hydro­philic sheets parallel to (001) (Fig. 2 ▸ *a*,*b*). Within a sheet, the coordinating water mol­ecules are solely engaged as donor groups whereas the non-coordinating water mol­ecules (O*w*1 and O*w*2 in (I) and (III), and O*w*1 in (II)) have the functions both as donor and acceptor groups. The carboxyl­ate acceptor atoms O6 and O7 in the structure of (I) and (III) and the pair of equivalent atoms O5 (*x*, *y*, *z* and 1 − *x*, *y*, *z*) in the structure of (II) stem from the second, non-coord­inating carboxyl­ate anion. Each of these carboxyl­ate oxygen atoms is an acceptor of three hydrogen bonds that are donated by two coordinating and by one non-coordinating water mol­ecules. Numerical values of these inter­actions are collated in Tables 4 ▸, 5 ▸ and 6 ▸ for structures (I), (II) and (III)), respectively. Fig. 3 ▸ *a*,*b* depict the hydrogen-bonded substructures in (II) and (III). The graph-set motifs are 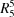
(10) (Etter *et al.*, 1990 ▸), which include these atoms: O*w*1–O*w*1^xiii^(−*x* + 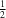, *y*, −*z* + 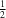)–O*w*1^xiv^(*x* + 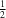, *y*, −*z* + 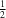)–O*w*1^iii^(−*x* + 1, *y*, *z*)–O3^xv^(−*x* + 1, *y* + 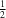, −*z* + 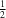) for (II) and O*w*1–O*w*2^v^(−*x* − 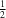, *y*, −*z* + 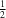)–O*w*1^ii^(*x* + 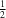, *y*, −*z* + 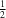)–O*w*2–O3^vi^(−*x* + 1, *y* + 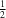, −*z* + 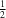) for (III), respectively. Note the disorder of the hydrogen atoms H2*ow*1 and H3*ow*1 bound to O*w*1 in the structure of (II).

The third type of cohesion force is related to van der Waals inter­actions between the hydro­phobic parts of the layers involving the methyl­ene and methyl groups. The shortest C⋯C distances observed in (I) and (III) are C4⋯C7(*x* + 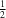, −*y*, *z* + 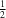), which are 3.762 (2) and 3.746 (2) Å, respectively. The shortest C⋯C inter­actions in (II) for C3*b*⋯C6 (*x* + 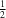, −*y* + 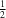, −*z*) and C3*b*⋯C7 (−*x* + 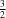, −*y* + 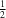, −*z*) are 3.569 (4) and 3.146 (5) Å, respectively. These comparatively shorter distances indicate positional disorder (see *Refinement* section). As a general rule, it can be inferred that the shorter the C⋯C distances between adjacent groups, the greater is the probability for the occurrence of positional disorder of the 1-methyl­ethyl group. See also the discussion regarding the observed disorder in barium dicalcium hexa­kis­(propano­ate) (CABAPN) by Stadnicka & Glazer (1980 ▸) where, however, the methyl carbons get as close as 4.05 (2) Å.

## Synthesis and crystallization   

For (III), two molar equivalents of CaCO_3_ and one molar equivalent of SrCO_3_ were neutralized by six molar equivalents of 2-methyl­propionic acid (using 0.76 g of CaCO_3_, 0.56 g of SrCO_3_ and about 2.50 g of 2-methyl­propionic acid). The solution was heated at 343 K, an excess of the acid was then added until the pH was between 5 and 6. The solution was filtered and then heated at 313 K until needle-like colourless crystals appeared. The pure Ca compound, (I), and the pure Sr compound, (II), were prepared for the sake of comparison. 0.85 g of CaCO_3_ were neutralized by 1.5 g of 2-methyl­propionic acid and 1.26 g of SrCO_3_ were neutralized by 1.5 g of 2-methyl­propionic acid, respectively; in each case these values correspond to the molar ratio of 1:2. The solutions were heated at 343 K, an excess of the acid was then added until the pH was between 5 and 6. The solutions were filtered and then heated at 313 K until needle-like colourless crystals appeared.

We have also tried to prepare magnesium 2-methyl­propano­ate and barium 2-methyl­propano­ate in a similar way as for (I)–(III). However, it turned out that the obtained crystals of the former compound correspond to VIQTOG, while the crystal structure of the latter compound is modulated and is being solved at present. Provided that we obtain a satisfactory model, the results will be published elsewhere.

## Structure determination and refinement   

Crystal data, data collection and structure refinement details are summarized in Table 7 ▸. In all structures, the methane­triyl hydrogen atoms were placed in calculated positions and refined with C_methane­tri­yl_—H_methane­tri­yl_ = 1.00 Å, *U*_iso_(H_methane­tri­yl_) = 1.2*U*_eq_(C_methane­tri­yl_). Methyl hydrogen atoms were discernible in difference electron-density maps and were refined with C_meth­yl_—H_meth­yl_ = 0.98 Å, *U*_iso_(H_meth­yl_) = 1.5*U*_eq_(C_meth­yl_). Finally, difference electron density maps revealed the water hydrogen atoms, which were refined with restraints of O_water_—H_water_ = 0.840 (1) Å.

For (II), difference electron-density maps revealed positional disorder of the 2-methyl­propyl entity in both anions. This positional disorder affects the non-oxygen atoms that are not situated on the mirror plane (Wyckoff position 8*f*). In addition, methyl atoms C3*a* and C3*b* with their attached hydrogen atoms were first subjected to a trial refinement of their occupancies, which resulted in 0.510 (5) and 0.490 (5) for C3*a* and C3*b* and the attached hydrogen atoms, respectively. In the final model, the occupancies were fixed at 0.50 for these groups. O*w*1 is situated in a general position like its hydrogen atoms. As a result of the local environment, H1*ow*1 was assumed to be fully occupied while H2*ow*1 and H3*ow*1 were supposed to be equally disordered over two positions. This assumption turned out to be in agreement with a trial refinement of their occupational parameters despite the very low scattering power of the hydrogen atoms.

For (III), the Ca/Sr occupation was refined [ratio 0.7936 (16):0.2064 (16)] under the assumption of the same position and the same displacement parameters for Ca and Sr and a fully occupied site. A B-C type 1 Lorentzian isotropic (Becker & Coppens, 1974 ▸) extinction correction was applied.

## Supplementary Material

Crystal structure: contains datablock(s) global, I, II, III. DOI: 10.1107/S2056989020012888/wm5584sup1.cif

Structure factors: contains datablock(s) I. DOI: 10.1107/S2056989020012888/wm5584Isup2.hkl

Supporting information file. DOI: 10.1107/S2056989020012888/wm5584Isup5.smi

Structure factors: contains datablock(s) II. DOI: 10.1107/S2056989020012888/wm5584IIsup3.hkl

Supporting information file. DOI: 10.1107/S2056989020012888/wm5584IIsup6.smi

Structure factors: contains datablock(s) III. DOI: 10.1107/S2056989020012888/wm5584IIIsup4.hkl

Supporting information file. DOI: 10.1107/S2056989020012888/wm5584IIIsup7.smi

CCDC references: 2033191, 2033190, 2033189

Additional supporting information:  crystallographic information; 3D view; checkCIF report

## Figures and Tables

**Table 1:** Selected bond lengths (Å) for (I)

Ca1—O3	2.3479 (12)	Ca1—O2	2.4993 (11)
Ca1—O2^i^	2.3653 (10)	Ca1—O1	2.5308 (11)
Ca1—O1^ii^	2.3938 (10)	Ca1—O4^ii^	2.5446 (11)
Ca1—O5	2.4085 (12)	Ca1—O4	2.6019 (11)

Symmetry codes: (i) 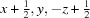; (ii) 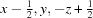.

**Table 2:** Selected bond lengths (Å) for (II)

Sr1—O1^i^	2.4788 (10)	Sr1—O1	2.6561 (11)
Sr1—O1^ii^	2.4788 (10)	Sr1—O1^iii^	2.6561 (11)
Sr1—O3	2.4899 (16)	Sr1—O2	2.6796 (11)
Sr1—O4	2.5593 (18)	Sr1—O2^ii^	2.6796 (11)

Symmetry codes: (i) 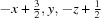; (ii) 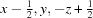; (iii) 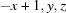.

**Table 3:** Selected bond lengths (Å) for (III)

Ca1—O3	2.3719 (10)	Ca1—O2	2.5457 (9)
Ca1—O2^i^	2.3845 (9)	Ca1—O1	2.5714 (9)
Ca1—O1^ii^	2.4091 (8)	Ca1—O4^ii^	2.5747 (9)
Ca1—O5	2.4209 (10)	Ca1—O4	2.6271 (9)

Symmetry codes: (i) 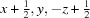; (ii) 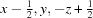.

**Table 4:** Hydrogen-bond geometry (Å, °) for (I)

*D*—H⋯*A*	*D*—H	H⋯*A*	*D*⋯*A*	*D*—H⋯*A*
O3—H1*o*3⋯O*w*2^iii^	0.840 (13)	2.063 (15)	2.8754 (16)	162.5 (15)
O3—H2*o*3⋯O*w*1^iii^	0.840 (14)	1.954 (15)	2.7829 (17)	168.8 (15)
O4—H1*o*4⋯O6	0.840 (13)	1.936 (13)	2.7560 (15)	165.1 (14)
O4—H2*o*4⋯O7^i^	0.840 (14)	1.925 (13)	2.7431 (15)	164.4 (14)
O5—H1*o*5⋯O6^ii^	0.840 (13)	1.966 (13)	2.7805 (15)	163.0 (18)
O5—H2*o*5⋯O7^i^	0.840 (13)	1.935 (13)	2.7597 (15)	166.9 (18)
O*w*1—H1*ow*1⋯O*w*2^iv^	0.840 (5)	1.882 (4)	2.7192 (17)	174 (2)
O*w*1—H2*ow*1⋯O7	0.840 (13)	1.960 (13)	2.7872 (17)	168.0 (18)
O*w*2—H1*ow*2⋯O6	0.840 (13)	1.969 (13)	2.8070 (17)	175.1 (17)
O*w*2—H2*ow*2⋯O*w*1^i^	0.840 (7)	1.886 (7)	2.7242 (18)	175 (2)

Symmetry codes: (i) 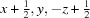; (ii) 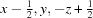; (iii) 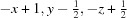; (iv) 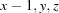.

**Table 5:** Hydrogen-bond geometry (Å, °) for (II)

*D*—H⋯*A*	*D*—H	H⋯*A*	*D*⋯*A*	*D*—H⋯*A*
O2—H1*o*2⋯O5	0.820 (16)	1.936 (15)	2.7465 (14)	169.3 (17)
O3—H1*o*3⋯O*w*1^iv^	0.827 (17)	2.022 (17)	2.8339 (18)	167.1 (18)
O4—H1*o*4⋯O5^i^	0.844 (18)	1.964 (17)	2.7887 (16)	165.3 (19)
O*w*1—H1*ow*1⋯O5^iii^	0.818 (18)	1.976 (19)	2.7913 (18)	174 (2)
O*w*1—H2*ow*1⋯O*w*1^v^	0.82 (2)	1.92 (3)	2.736 (2)	176 (5)
O*w*1—H3*ow*1⋯O*w*1^vi^	0.803 (17)	1.946 (17)	2.747 (2)	176 (5)

Symmetry codes: (i) 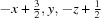; (iii) 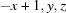; (iv) 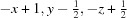; (v) 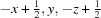; (vi) 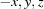.

**Table 6:** Hydrogen-bond geometry (Å, °) for (III)

*D*—H⋯*A*	*D*—H	H⋯*A*	*D*⋯*A*	*D*—H⋯*A*
O3—H1*o*3⋯O*w*2^iii^	0.840 (11)	2.061 (12)	2.8767 (14)	163.6 (13)
O3—H2*o*3⋯O*w*1^iii^	0.840 (12)	1.953 (12)	2.7842 (14)	169.8 (13)
O4—H1*o*4⋯O6	0.840 (11)	1.932 (10)	2.7498 (13)	164.2 (11)
O4—H2*o*4⋯O7^i^	0.840 (11)	1.920 (10)	2.7382 (13)	164.2 (11)
O5—H1*o*5⋯O6^ii^	0.840 (11)	1.964 (11)	2.7831 (13)	164.9 (15)
O5—H2*o*5⋯O7^i^	0.840 (11)	1.944 (11)	2.7636 (13)	165.0 (14)
O*w*1—H1*ow*1⋯O*w*2^iv^	0.840 (4)	1.888 (3)	2.7233 (14)	172.8 (16)
O*w*1—H2*ow*1⋯O7	0.840 (10)	1.951 (11)	2.7836 (14)	170.7 (15)
O*w*2—H1*ow*2⋯O6	0.840 (10)	1.967 (10)	2.8050 (14)	175.3 (15)
O*w*2—H2*ow*2⋯O*w*1^i^	0.840 (5)	1.887 (5)	2.7248 (15)	175.2 (17)

Symmetry codes: (i) 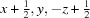; (ii) 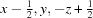; (iii) 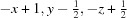; (iv) 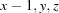.

**Table 7:** Experimental details

	(I)	(II)	(III)
Crystal data			
Chemical formula	[Ca(C_4_H_7_O_2_)(H_2_O)_3_]·C_4_H_7_O_2_·2H_2_O	[Sr(C_4_H_7_O_2_)(H_2_O)_3_]·C_4_H_7_O_2_·2H_2_O	[Ca_0.794_Sr_0.206_(C_4_H_7_O_2_)(H_2_O)_3_]·C_4_H_7_O_2_·2H_2_O
*M*_r_	304.4	351.9	314.2
Crystal system, space group	Orthorhombic, *P* *b* *c* *a*	Orthorhombic, *C* *m* *c* *e*	Orthorhombic, *P* *b* *c* *a*
Temperature (K)	120	120	120
*a*, *b*, *c* (Å)	6.6662 (2), 19.5903 (7), 23.4286 (8)	6.8801 (3), 19.7520 (11), 23.2734 (13)	6.7153 (3), 19.6061 (10), 23.3498 (11)
*V* (Å^3^)	3059.61 (18)	3162.8 (3)	3074.3 (3)
*Z*	8	8	8
Radiation type	Mo *K*α	Mo *K*α	Mo *K*α
μ (mm^−1^)	0.44	3.44	1.08
Crystal size (mm)	0.27 × 0.17 × 0.05	0.24 × 0.12 × 0.08	0.59 × 0.18 × 0.08

Data collection			
Diffractometer	Bruker D8 VENTURE Kappa Duo PHOTON 100 CMOS	Bruker D8 VENTURE Kappa Duo PHOTON 100 CMOS	Bruker D8 VENTURE Kappa Duo PHOTON 100 CMOS
Absorption correction	Multi-scan (*SADABS*; Bruker, 2017 ▸)	Multi-scan (*SADABS*; Bruker, 2017 ▸)	Multi-scan (*SADABS*; Bruker, 2017 ▸)
*T*_min_, *T*_max_	0.889, 0.980	0.491, 0.770	0.573, 0.917
No. of measured, independent and observed [*I* > 3σ(*I*)] reflections	25865, 3511, 2955	21213, 1965, 1762	25462, 3533, 2787
*R*_int_	0.033	0.032	0.030
(sin θ/λ)_max_ (Å^−1^)	0.649	0.649	0.651

Refinement			
*R*[*F* > 3σ(*F*)], *wR*(*F*), *S*	0.039, 0.081, 1.99	0.021, 0.055, 1.79	0.026, 0.062, 1.54
No. of reflections	3511	1965	3533
No. of parameters	193	126	195
No. of restraints	10	9	10
H-atom treatment	H atoms treated by a mixture of independent and constrained refinement	H atoms treated by a mixture of independent and constrained refinement	H atoms treated by a mixture of independent and constrained refinement
Δρ_max_, Δρ_min_ (e Å^−3^)	0.31, −0.38	0.37, −0.30	0.27, −0.25

Computer programs: *APEX2* and *SAINT* (Bruker, 2017 ▸), *SUPERFLIP* (Palatinus & Chapuis, 2007 ▸), *SHELXT* (Sheldrick, 2015 ▸), *JANA*2006 (Petříček *et al.*, 2014 ▸), *DIAMOND* (Brandenburg, 2015 ▸) and *publCIF* (Westrip, 2010 ▸).

## supplementary crystallographic information

### \ catena-Poly[[µ-aqua-diaqua(µ3-2-methylpropanoato-\ κ4O:O,O':O')calcium] 2-methylpropanoate dihydrate] (I) Crystal data

**Table:**

[Ca(C_4_H_7_O_2_)(H_2_O)_3_]·C_4_H_7_O_2_·2H_2_O	There have been used diffractions with I/σ(I)>20 for the unit cell determination.
*M**_r_* = 304.4	*D*_x_ = 1.321 Mg m^−^^3^
Orthorhombic, *P**b**c**a*	Mo *K*α radiation, λ = 0.71073 Å
Hall symbol: -P 2ac 2ab	Cell parameters from 9916 reflections
*a* = 6.6662 (2) Å	θ = 2.3–27.5°
*b* = 19.5903 (7) Å	µ = 0.44 mm^−^^1^
*c* = 23.4286 (8) Å	*T* = 120 K
*V* = 3059.61 (18) Å^3^	Prism, colourless
*Z* = 8	0.27 × 0.17 × 0.05 mm
*F*(000) = 1312	

### \ catena-Poly[[µ-aqua-diaqua(µ3-2-methylpropanoato-\ κ4O:O,O':O')calcium] 2-methylpropanoate dihydrate] (I) Data collection

**Table:**

Bruker D8 VENTURE Kappa Duo PHOTON 100 CMOS diffractometer	3511 independent reflections
Radiation source: X-ray tube	2955 reflections with *I* > 3σ(*I*)
Quazar Mo multilayer optic monochromator	*R*_int_ = 0.033
φ and ω scans	θ_max_ = 27.5°, θ_min_ = 2.1°
Absorption correction: multi-scan (*SADABS*; Bruker, 2017)	*h* = −8→8
*T*_min_ = 0.889, *T*_max_ = 0.980	*k* = −24→25
25865 measured reflections	*l* = −30→30

### \ catena-Poly[[µ-aqua-diaqua(µ3-2-methylpropanoato-\ κ4O:O,O':O')calcium] 2-methylpropanoate dihydrate] (I) Refinement

**Table:**

Refinement on *F*^2^	66 constraints
*R*[*F* > 3σ(*F*)] = 0.039	Primary atom site location: charge flipping
*wR*(*F*) = 0.081	H atoms treated by a mixture of independent and constrained refinement
*S* = 1.99	Weighting scheme based on measured s.u.'s *w* = 1/(σ^2^(*I*) + 0.0004*I*^2^)
3511 reflections	(Δ/σ)_max_ = 0.015
193 parameters	Δρ_max_ = 0.31 e Å^−^^3^
10 restraints	Δρ_min_ = −0.38 e Å^−^^3^

### \ catena-Poly[[µ-aqua-diaqua(µ3-2-methylpropanoato-\ κ4O:O,O':O')calcium] 2-methylpropanoate dihydrate] (I) Fractional atomic coordinates and isotropic or equivalent isotropic displacement parameters (Å^2^)

**Table:**

	*x*	*y*	*z*	*U*_iso_*/*U*_eq_	
Ca1	0.51243 (4)	0.148107 (15)	0.283542 (12)	0.01153 (9)	
O1	0.67506 (14)	0.12357 (6)	0.18811 (5)	0.0155 (3)	
O2	0.34606 (14)	0.12092 (6)	0.19069 (4)	0.0161 (3)	
C1	0.5086 (2)	0.11831 (8)	0.16303 (6)	0.0129 (4)	
C2	0.4989 (2)	0.10975 (8)	0.09862 (7)	0.0165 (4)	
H1c2	0.446418	0.153868	0.083208	0.0198*	
C3	0.3552 (3)	0.05243 (10)	0.08221 (7)	0.0284 (6)	
H1c3	0.349227	0.048592	0.040536	0.0426*	
H2c3	0.403026	0.009271	0.098419	0.0426*	
H3c3	0.221179	0.06258	0.097119	0.0426*	
C4	0.7039 (2)	0.09872 (10)	0.07130 (7)	0.0238 (5)	
H1c4	0.689212	0.096932	0.029701	0.0357*	
H2c4	0.793135	0.136497	0.081634	0.0357*	
H3c4	0.761063	0.055638	0.084979	0.0357*	
O3	0.49949 (16)	0.03175 (6)	0.30728 (6)	0.0229 (4)	
H1o3	0.3920 (16)	0.0098 (9)	0.3109 (8)	0.0343*	
H2o3	0.594 (2)	0.0035 (8)	0.3078 (8)	0.0343*	
O4	0.76674 (16)	0.23978 (6)	0.24860 (5)	0.0156 (3)	
H1o4	0.737 (3)	0.2657 (8)	0.2213 (5)	0.0233*	
H2o4	0.804 (3)	0.2649 (8)	0.2756 (5)	0.0233*	
O5	0.52665 (15)	0.22166 (6)	0.36582 (5)	0.0168 (3)	
H1o5	0.4273 (17)	0.2477 (8)	0.3694 (8)	0.0252*	
H2o5	0.6286 (17)	0.2468 (8)	0.3667 (8)	0.0252*	
O6	0.69414 (15)	0.30599 (6)	0.14688 (4)	0.0181 (3)	
O7	0.36106 (15)	0.30246 (6)	0.15020 (5)	0.0180 (3)	
C5	0.5238 (2)	0.31076 (8)	0.12347 (6)	0.0137 (4)	
C6	0.5131 (2)	0.33159 (9)	0.06102 (7)	0.0179 (4)	
H1c6	0.379623	0.317605	0.045454	0.0215*	
C7	0.5390 (3)	0.40883 (9)	0.05774 (7)	0.0268 (5)	
H1c7	0.530688	0.42353	0.017824	0.0403*	
H2c7	0.432769	0.431102	0.079868	0.0403*	
H3c7	0.670091	0.421507	0.073409	0.0403*	
C8	0.6681 (3)	0.29543 (10)	0.02414 (7)	0.0295 (6)	
H1c8	0.651725	0.309588	−0.015704	0.0443*	
H2c8	0.803143	0.307466	0.037273	0.0443*	
H3c8	0.649309	0.245941	0.02711	0.0443*	
Ow1	0.22518 (17)	0.42507 (7)	0.19654 (5)	0.0225 (4)	
H1ow1	0.1005 (5)	0.4296 (11)	0.1931 (8)	0.0337*	
H2ow1	0.253 (3)	0.3889 (6)	0.1790 (8)	0.0337*	
Ow2	0.81843 (18)	0.43259 (7)	0.19043 (5)	0.0229 (4)	
H1ow2	0.782 (3)	0.3957 (5)	0.1754 (8)	0.0344*	
H2ow2	0.791 (3)	0.4276 (11)	0.2252 (2)	0.0344*	

### \ catena-Poly[[µ-aqua-diaqua(µ3-2-methylpropanoato-\ κ4O:O,O':O')calcium] 2-methylpropanoate dihydrate] (I) Atomic displacement parameters (Å^2^)

**Table:**

	*U*^11^	*U*^22^	*U*^33^	*U*^12^	*U*^13^	*U*^23^
Ca1	0.00793 (14)	0.01282 (15)	0.01385 (16)	0.00014 (12)	−0.00009 (12)	−0.00034 (11)
O1	0.0097 (5)	0.0190 (6)	0.0176 (6)	−0.0006 (4)	−0.0018 (4)	−0.0019 (5)
O2	0.0099 (5)	0.0213 (6)	0.0173 (6)	0.0007 (5)	0.0010 (4)	−0.0039 (5)
C1	0.0124 (7)	0.0089 (7)	0.0175 (7)	0.0007 (6)	−0.0003 (6)	0.0006 (6)
C2	0.0153 (7)	0.0187 (8)	0.0154 (7)	−0.0004 (7)	−0.0001 (6)	0.0011 (6)
C3	0.0300 (10)	0.0383 (12)	0.0169 (9)	−0.0148 (9)	−0.0002 (7)	−0.0072 (8)
C4	0.0217 (8)	0.0315 (11)	0.0182 (9)	−0.0031 (8)	0.0042 (7)	−0.0065 (8)
O3	0.0121 (5)	0.0141 (6)	0.0424 (7)	0.0006 (5)	0.0006 (5)	0.0027 (5)
O4	0.0172 (5)	0.0150 (6)	0.0145 (6)	0.0002 (5)	−0.0009 (4)	0.0004 (5)
O5	0.0114 (5)	0.0183 (6)	0.0207 (6)	−0.0007 (4)	0.0009 (5)	−0.0028 (5)
O6	0.0151 (5)	0.0217 (7)	0.0175 (6)	0.0018 (5)	−0.0029 (4)	0.0010 (5)
O7	0.0142 (5)	0.0200 (6)	0.0198 (6)	−0.0016 (5)	0.0029 (4)	0.0009 (5)
C5	0.0160 (7)	0.0078 (7)	0.0173 (7)	0.0001 (6)	0.0002 (6)	−0.0013 (6)
C6	0.0149 (7)	0.0217 (8)	0.0170 (8)	−0.0021 (7)	−0.0017 (6)	0.0014 (6)
C7	0.0380 (10)	0.0210 (9)	0.0215 (9)	0.0040 (8)	−0.0008 (8)	0.0061 (7)
C8	0.0419 (11)	0.0276 (11)	0.0191 (9)	0.0059 (9)	0.0078 (8)	0.0003 (8)
Ow1	0.0164 (6)	0.0178 (7)	0.0333 (7)	0.0000 (5)	0.0021 (5)	−0.0035 (5)
Ow2	0.0213 (6)	0.0194 (7)	0.0280 (7)	0.0000 (5)	−0.0028 (5)	−0.0035 (6)

### \ catena-Poly[[µ-aqua-diaqua(µ3-2-methylpropanoato-\ κ4O:O,O':O')calcium] 2-methylpropanoate dihydrate] (I) Geometric parameters (Å, º)

**Table:**

Ca1—O3	2.3479 (12)	C8—H1c8	0.98
Ca1—O2^i^	2.3653 (10)	C8—H2c8	0.98
Ca1—O1^ii^	2.3938 (10)	C8—H3c8	0.98
Ca1—O5	2.4085 (12)	O3—H1o3	0.840 (13)
Ca1—O2	2.4993 (11)	O3—H2o3	0.840 (14)
Ca1—O1	2.5308 (11)	O4—H1o4	0.840 (13)
Ca1—O4^ii^	2.5446 (11)	O4—H2o4	0.840 (14)
Ca1—O4	2.6019 (11)	O5—H1o5	0.840 (13)
O1—C1	1.2597 (17)	O5—H2o5	0.840 (13)
O2—C1	1.2637 (17)	Ow1—H1ow1	0.840 (5)
C1—C2	1.520 (2)	Ow1—H2ow1	0.840 (13)
C2—H1c2	1	Ow2—H1ow2	0.840 (13)
C2—C3	1.525 (2)	Ow2—H2ow2	0.840 (7)
C2—C4	1.524 (2)	C2—C6	4.435 (2)
C3—H1c3	0.98	C2—C8	4.189 (2)
C3—H2c3	0.98	C2—C8^iii^	4.073 (2)
C3—H3c3	0.98	C3—C4^iv^	4.443 (2)
C4—H1c4	0.98	C3—C7^iii^	3.971 (2)
C4—H2c4	0.98	C3—C7^v^	3.892 (3)
C4—H3c4	0.98	C3—C8^iii^	4.080 (3)
O6—C5	1.2643 (18)	C4—C6^vi^	3.965 (2)
O7—C5	1.2634 (18)	C4—C7^vi^	3.762 (2)
C5—C6	1.520 (2)	C4—C7^vii^	4.108 (3)
C6—H1c6	1	C4—C8	4.016 (3)
C6—C7	1.525 (2)	C4—C8^vi^	4.345 (2)
C6—C8	1.522 (2)	C6—C8^iii^	3.932 (2)
C7—H1c7	0.98	C8—C8^iii^	3.944 (3)
C7—H2c7	0.98	C8—C8^vi^	3.944 (3)
C7—H3c7	0.98		
			
O1—Ca1—O1^ii^	127.55 (4)	H1c2—C2—C4	106.53
O1—Ca1—O2	51.73 (3)	C3—C2—C4	110.65 (14)
O1—Ca1—O2^i^	77.29 (3)	C2—C3—H1c3	109.47
O1—Ca1—O3	92.33 (4)	C2—C3—H2c3	109.47
O1—Ca1—O4	64.79 (4)	C2—C3—H3c3	109.47
O1—Ca1—O4^ii^	98.53 (4)	H1c3—C3—H2c3	109.47
O1—Ca1—O5	143.49 (4)	H1c3—C3—H3c3	109.47
O1^ii^—Ca1—O2	77.40 (3)	H2c3—C3—H3c3	109.47
O1^ii^—Ca1—O2^i^	140.12 (4)	C2—C4—H1c4	109.47
O1^ii^—Ca1—O3	72.83 (4)	C2—C4—H2c4	109.47
O1^ii^—Ca1—O4	146.94 (4)	C2—C4—H3c4	109.47
O1^ii^—Ca1—O4^ii^	67.61 (4)	H1c4—C4—H2c4	109.47
O1^ii^—Ca1—O5	86.28 (4)	H1c4—C4—H3c4	109.47
O2—Ca1—O2^i^	126.26 (4)	H2c4—C4—H3c4	109.47
O2—Ca1—O3	89.03 (4)	C5—C6—H1c6	108.62
O2—Ca1—O4	99.34 (4)	C5—C6—C7	108.04 (13)
O2—Ca1—O4^ii^	66.87 (4)	C5—C6—C8	112.92 (13)
O2—Ca1—O5	147.31 (4)	H1c6—C6—C7	110.74
O2^i^—Ca1—O3	75.85 (4)	H1c6—C6—C8	105.64
O2^i^—Ca1—O4	67.86 (4)	C7—C6—C8	110.89 (14)
O2^i^—Ca1—O4^ii^	147.11 (4)	C6—C7—H1c7	109.47
O2^i^—Ca1—O5	83.88 (4)	C6—C7—H2c7	109.47
O3—Ca1—O4	140.23 (4)	C6—C7—H3c7	109.47
O3—Ca1—O4^ii^	137.03 (4)	H1c7—C7—H2c7	109.47
O3—Ca1—O5	113.12 (4)	H1c7—C7—H3c7	109.47
O4—Ca1—O4^ii^	80.74 (3)	H2c7—C7—H3c7	109.47
O4—Ca1—O5	79.23 (4)	C6—C8—H1c8	109.47
O4^ii^—Ca1—O5	80.79 (4)	C6—C8—H2c8	109.47
Ca1—O1—Ca1^i^	96.85 (4)	C6—C8—H3c8	109.47
Ca1—O2—Ca1^ii^	98.46 (4)	H1c8—C8—H2c8	109.47
Ca1—O4—Ca1^i^	91.45 (4)	H1c8—C8—H3c8	109.47
O1—C1—O2	120.85 (13)	H2c8—C8—H3c8	109.47
O1—C1—C2	120.64 (12)	H1o3—O3—H2o3	107.6 (15)
O2—C1—C2	118.50 (12)	H1o4—O4—H2o4	106.8 (14)
C1—C2—H1c2	106.15	H1o5—O5—H2o5	106.3 (14)
C1—C2—C3	111.00 (13)	H1ow1—Ow1—H2ow1	105 (2)
C1—C2—C4	113.24 (12)	H1ow2—Ow2—H2ow2	104.1 (19)
H1c2—C2—C3	108.99		

Symmetry codes: (i) *x*+1/2, *y*, −*z*+1/2; (ii) *x*−1/2, *y*, −*z*+1/2; (iii) *x*−1/2, −*y*+1/2, −*z*; (iv) *x*−1, *y*, *z*; (v) −*x*+1/2, *y*−1/2, *z*; (vi) *x*+1/2, −*y*+1/2, −*z*; (vii) −*x*+3/2, *y*−1/2, *z*.

### \ catena-Poly[[µ-aqua-diaqua(µ3-2-methylpropanoato-\ κ4O:O,O':O')calcium] 2-methylpropanoate dihydrate] (I) Hydrogen-bond geometry (Å, º)

**Table:**

*D*—H···*A*	*D*—H	H···*A*	*D*···*A*	*D*—H···*A*
O3—H1*o*3···O*w*2^viii^	0.840 (13)	2.063 (15)	2.8754 (16)	162.5 (15)
O3—H2*o*3···O*w*1^viii^	0.840 (14)	1.954 (15)	2.7829 (17)	168.8 (15)
O4—H1*o*4···O6	0.840 (13)	1.936 (13)	2.7560 (15)	165.1 (14)
O4—H2*o*4···O7^i^	0.840 (14)	1.925 (13)	2.7431 (15)	164.4 (14)
O5—H1*o*5···O6^ii^	0.840 (13)	1.966 (13)	2.7805 (15)	163.0 (18)
O5—H2*o*5···O7^i^	0.840 (13)	1.935 (13)	2.7597 (15)	166.9 (18)
O*w*1—H1*ow*1···O*w*2^iv^	0.840 (5)	1.882 (4)	2.7192 (17)	174 (2)
O*w*1—H2*ow*1···O7	0.840 (13)	1.960 (13)	2.7872 (17)	168.0 (18)
O*w*2—H1*ow*2···O6	0.840 (13)	1.969 (13)	2.8070 (17)	175.1 (17)
O*w*2—H2*ow*2···O*w*1^i^	0.840 (7)	1.886 (7)	2.7242 (18)	175 (2)

Symmetry codes: (i) *x*+1/2, *y*, −*z*+1/2; (ii) *x*−1/2, *y*, −*z*+1/2; (iv) *x*−1, *y*, *z*; (viii) −*x*+1, *y*−1/2, −*z*+1/2.

### catena-Poly[[µ-aqua-diaqua(µ3-2-methylpropanoato-\ κ4O:O,O':O')strontium] 2-methylpropanoate dihydrate] (II) Crystal data

**Table:**

[Sr(C_4_H_7_O_2_)(H_2_O)_3_]·C_4_H_7_O_2_·2H_2_O	There have been used diffractions with I/σ(I)>20 for the unit cell determination.
*M**_r_* = 351.9	*D*_x_ = 1.478 Mg m^−^^3^
Orthorhombic, *C**m**c**e*	Mo *K*α radiation, λ = 0.71073 Å
Hall symbol: -C 2bc 2	Cell parameters from 9972 reflections
*a* = 6.8801 (3) Å	θ = 2.2–27.5°
*b* = 19.7520 (11) Å	µ = 3.44 mm^−^^1^
*c* = 23.2734 (13) Å	*T* = 120 K
*V* = 3162.8 (3) Å^3^	Prism, colourless
*Z* = 8	0.24 × 0.12 × 0.08 mm
*F*(000) = 1456	

### catena-Poly[[µ-aqua-diaqua(µ3-2-methylpropanoato-\ κ4O:O,O':O')strontium] 2-methylpropanoate dihydrate] (II) Data collection

**Table:**

Bruker D8 VENTURE Kappa Duo PHOTON 100 CMOS diffractometer	1965 independent reflections
Radiation source: X-ray tube	1762 reflections with *I* > 3σ(*I*)
Quazar Mo multilayer optic monochromator	*R*_int_ = 0.032
φ and ω scans	θ_max_ = 27.5°, θ_min_ = 2.2°
Absorption correction: multi-scan (*SADABS*; Bruker, 2017)	*h* = −8→8
*T*_min_ = 0.491, *T*_max_ = 0.770	*k* = −25→25
21213 measured reflections	*l* = −30→27

### catena-Poly[[µ-aqua-diaqua(µ3-2-methylpropanoato-\ κ4O:O,O':O')strontium] 2-methylpropanoate dihydrate] (II) Refinement

**Table:**

Refinement on *F*^2^	62 constraints
*R*[*F* > 3σ(*F*)] = 0.021	Primary atom site location: dual-space method
*wR*(*F*) = 0.055	H atoms treated by a mixture of independent and constrained refinement
*S* = 1.79	Weighting scheme based on measured s.u.'s *w* = 1/(σ^2^(*I*) + 0.0004*I*^2^)
1965 reflections	(Δ/σ)_max_ = 0.050
126 parameters	Δρ_max_ = 0.37 e Å^−^^3^
9 restraints	Δρ_min_ = −0.30 e Å^−^^3^

### catena-Poly[[µ-aqua-diaqua(µ3-2-methylpropanoato-\ κ4O:O,O':O')strontium] 2-methylpropanoate dihydrate] (II) Special details

**Table:**

Refinement. The positions of the methyl hydrogen atoms s H1C6, H2C6 and H3C6 were restrained by the angle restraints 56.250?(1) ° for the angles H1c6—C6—H2c6iii, H2c6—C6—H2c6iii, H2c6—C6—H2c6iii, H3c6—C6—H3c6iii ((iii) x + 1, y, z).

### catena-Poly[[µ-aqua-diaqua(µ3-2-methylpropanoato-\ κ4O:O,O':O')strontium] 2-methylpropanoate dihydrate] (II) Fractional atomic coordinates and isotropic or equivalent isotropic displacement parameters (Å^2^)

**Table:**

	*x*	*y*	*z*	*U*_iso_*/*U*_eq_	Occ. (<1)
Sr1	0.5	0.145526 (9)	0.287511 (8)	0.01228 (6)	
O1	0.65991 (15)	0.11785 (5)	0.18632 (5)	0.0166 (3)	
C1	0.5	0.11399 (10)	0.15989 (9)	0.0130 (6)	
C2	0.5	0.10609 (11)	0.09535 (10)	0.0163 (6)	
H1c2	0.44623	0.149367	0.079711	0.0196*	0.5
C3a	0.3722 (5)	0.04613 (19)	0.07858 (15)	0.0299 (11)	0.5
H1c3a	0.429281	0.004234	0.093548	0.0448*	0.5
H2c3a	0.241896	0.052214	0.094806	0.0448*	0.5
H3c3a	0.363233	0.043419	0.036618	0.0448*	0.5
C3b	0.7010 (5)	0.09931 (18)	0.06811 (14)	0.0233 (10)	0.5
H1c3b	0.778228	0.139682	0.076891	0.035*	0.5
H2c3b	0.766218	0.059155	0.08359	0.035*	0.5
H3c3b	0.687385	0.094733	0.026376	0.035*	0.5
O2	0.75	0.23969 (8)	0.25	0.0159 (4)	
H1o2	0.717 (3)	0.2627 (9)	0.2223 (6)	0.0238*	
O3	0.5	0.02368 (8)	0.31496 (8)	0.0218 (5)	
H1o3	0.599 (2)	−0.0003 (10)	0.3152 (9)	0.0328*	
O4	0.5	0.22986 (9)	0.37100 (8)	0.0276 (6)	
H1o4	0.597 (2)	0.2558 (10)	0.3707 (9)	0.0414*	
O5	0.66149 (19)	0.30397 (6)	0.14868 (5)	0.0267 (4)	
C4	0.5	0.31058 (11)	0.12363 (10)	0.0204 (7)	
C5	0.5	0.33102 (9)	0.06050 (8)	0.0185 (6)	
H1c5	0.378186	0.314569	0.041672	0.0222*	0.5
C6	0.5	0.40764 (9)	0.05703 (8)	0.0570 (13)	
H1c6	0.365706	0.424153	0.056282	0.0855*	0.5
H2c6	0.56715	0.426313	0.090613	0.0855*	0.5
H3c6	0.567143	0.421994	0.021948	0.0855*	0.5
C7	0.6601 (5)	0.2941 (2)	0.02353 (15)	0.0302 (12)	0.5
H1c7	0.788041	0.313174	0.032479	0.0452*	0.5
H2c7	0.659677	0.245607	0.032529	0.0452*	0.5
H3c7	0.632122	0.300485	−0.017398	0.0452*	0.5
Ow1	0.1996 (2)	0.42598 (7)	0.19314 (6)	0.0323 (4)	
H1ow1	0.233 (3)	0.3890 (8)	0.1810 (10)	0.0484*	
H2ow1	0.228 (8)	0.424 (2)	0.2273 (9)	0.0484*	0.5
H3ow1	0.083 (2)	0.424 (2)	0.1927 (19)	0.0484*	0.5

### catena-Poly[[µ-aqua-diaqua(µ3-2-methylpropanoato-\ κ4O:O,O':O')strontium] 2-methylpropanoate dihydrate] (II) Atomic displacement parameters (Å^2^)

**Table:**

	*U*^11^	*U*^22^	*U*^33^	*U*^12^	*U*^13^	*U*^23^
Sr1	0.01072 (11)	0.01255 (11)	0.01357 (12)	0	0	−0.00006 (8)
O1	0.0105 (5)	0.0232 (6)	0.0160 (6)	0.0002 (4)	−0.0021 (4)	−0.0048 (5)
C1	0.0139 (10)	0.0088 (9)	0.0163 (11)	0	0	−0.0014 (8)
C2	0.0160 (11)	0.0193 (11)	0.0137 (11)	0	0	0.0003 (9)
C3a	0.0282 (19)	0.044 (2)	0.0176 (17)	−0.0197 (17)	0.0033 (15)	−0.0109 (16)
C3b	0.0209 (16)	0.0328 (19)	0.0164 (16)	−0.0053 (14)	0.0062 (13)	−0.0033 (14)
O2	0.0209 (8)	0.0128 (7)	0.0140 (8)	0	−0.0028 (7)	0
O3	0.0114 (8)	0.0127 (8)	0.0415 (11)	0	0	0.0013 (7)
O4	0.0419 (12)	0.0215 (9)	0.0194 (9)	0	0	−0.0014 (7)
O5	0.0430 (8)	0.0199 (6)	0.0172 (6)	0.0072 (6)	−0.0088 (5)	0.0004 (5)
C4	0.0384 (14)	0.0081 (9)	0.0145 (11)	0	0	−0.0002 (8)
C5	0.0226 (12)	0.0198 (11)	0.0132 (11)	0	0	0.0012 (9)
C6	0.128 (3)	0.0216 (14)	0.0217 (15)	0	0	0.0094 (12)
C7	0.041 (2)	0.034 (2)	0.0162 (17)	0.0110 (17)	0.0050 (16)	−0.0005 (15)
Ow1	0.0426 (8)	0.0220 (7)	0.0322 (7)	−0.0113 (6)	0.0130 (7)	−0.0072 (6

### catena-Poly[[µ-aqua-diaqua(µ3-2-methylpropanoato-\ κ4O:O,O':O')strontium] 2-methylpropanoate dihydrate] (II) Geometric parameters (Å, º)

**Table:**

Sr1—O1^i^	2.4788 (10)	C5—C7^iii^	1.577 (4)
Sr1—O1^ii^	2.4788 (10)	C6—H1c6	0.98
Sr1—O3	2.4899 (16)	C6—H1c6^iii^	0.98
Sr1—O4	2.5593 (18)	C6—H2c6	0.98
Sr1—O1	2.6561 (11)	C6—H2c6^iii^	0.98
Sr1—O1^iii^	2.6561 (11)	C6—H3c6	0.98
Sr1—O2	2.6796 (11)	C6—H3c6^iii^	0.98
Sr1—O2^ii^	2.6796 (11)	C7—H1c7	0.98
O1—C1	1.2629 (15)	C7—H2c7	0.98
C1—C2	1.510 (3)	C7—H3c7	0.98
C2—H1c2	1	Ow1—H1ow1	0.818 (18)
C2—H1c2^iii^	1	Ow1—H2ow1	0.82 (2)
C2—C3a	1.526 (4)	Ow1—H3ow1	0.803 (17)
C2—C3a^iii^	1.526 (4)	C2—C7	4.217 (4)
C2—C3b	1.527 (3)	C2—C7^iv^	4.124 (4)
C2—C3b^iii^	1.527 (3)	C2—C7^v^	4.124 (4)
C3a—H1c3a	0.98	C2—C7^iii^	4.217 (4)
C3a—H2c3a	0.98	C3a—C3a^vi^	4.087 (5)
C3a—H3c3a	0.98	C3a—C3a^vii^	4.449 (5)
C3b—H1c3b	0.98	C3a—C3b^vii^	4.491 (5)
C3b—H2c3b	0.98	C3a—C6^viii^	3.781 (4)
C3b—H3c3b	0.98	C3a—C6^iv^	4.166 (4)
O2—H1o2	0.820 (16)	C3a—C7^iv^	4.212 (5)
O2—H1o2^i^	0.820 (16)	C3b—C3b^ix^	4.115 (5)
O3—H1o3	0.827 (17)	C3b—C5^x^	3.884 (4)
O3—H1o3^iii^	0.827 (17)	C3b—C6^xi^	4.317 (4)
O4—H1o4	0.844 (18)	C3b—C6^x^	3.569 (4)
O4—H1o4^iii^	0.844 (18)	C3b—C7	3.993 (5)
O5—C4	1.2615 (16)	C3b—C7^x^	4.356 (5)
C4—C5	1.524 (3)	C3b—C7^v^	3.146 (5)
C5—H1c5	1	C5—C7^iv^	3.923 (4)
C5—H1c5^iii^	1	C5—C7^v^	3.923 (4)
C5—C6	1.516 (3)	C7—C7^iv^	4.007 (5)
C5—C7	1.577 (4)	C7—C7^x^	4.007 (5)
			
O1—Sr1—O1^i^	77.38 (3)	C2—C3a—H3c3a	109.47
O1—Sr1—O1^ii^	124.29 (3)	H1c3a—C3a—H2c3a	109.47
O1—Sr1—O1^iii^	48.94 (3)	H1c3a—C3a—H3c3a	109.47
O1—Sr1—O2	65.67 (3)	H1c3a^iii^—C3a—H3c3a	103.72
O1—Sr1—O2^ii^	96.88 (2)	H2c3a—C3a—H3c3a	109.47
O1—Sr1—O3	91.64 (5)	C2—C3b—H1c3b	109.47
O1—Sr1—O4	143.81 (4)	C2—C3b—H2c3b	109.47
O1^i^—Sr1—O1^ii^	141.45 (4)	C2—C3b—H3c3b	109.47
O1^i^—Sr1—O1^iii^	124.29 (3)	H1c3b—C3b—H2c3b	109.47
O1^i^—Sr1—O2	68.11 (3)	H1c3b—C3b—H3c3b	109.47
O1^i^—Sr1—O2^ii^	147.04 (3)	H2c3b—C3b—H3c3b	109.47
O1^i^—Sr1—O3	73.97 (3)	H1o2—O2—H1o2^i^	112.6 (16)
O1^i^—Sr1—O4	87.54 (3)	H1o3—O3—H1o3^iii^	110.2 (17)
O1^ii^—Sr1—O1^iii^	77.38 (3)	H1o4—O4—H1o4^iii^	105.2 (18)
O1^ii^—Sr1—O2	147.04 (3)	O5—C4—O5^iii^	123.46 (19)
O1^ii^—Sr1—O2^ii^	68.11 (3)	O5—C4—C5	118.23 (10)
O1^ii^—Sr1—O3	73.97 (3)	O5^iii^—C4—C5	118.23 (10)
O1^ii^—Sr1—O4	87.54 (3)	C4—C5—H1c5	109.66
O1^iii^—Sr1—O2	96.88 (2)	C4—C5—H1c5^iii^	109.66
O1^iii^—Sr1—O2^ii^	65.67 (3)	C4—C5—C6	108.42 (15)
O1^iii^—Sr1—O3	91.64 (5)	C4—C5—C7	113.80 (17)
O1^iii^—Sr1—O4	143.81 (4)	C4—C5—C7^iii^	113.80 (17)
O2—Sr1—O2^ii^	79.87 (3)	H1c5—C5—H1c5^iii^	113.88
O2—Sr1—O3	138.98 (2)	H1c5—C5—C6	107.52
O2—Sr1—O4	78.21 (3)	C5—C6—H1c6	109.47
O2^ii^—Sr1—O3	138.98 (2)	C5—C6—H1c6^iii^	109.47
O2^ii^—Sr1—O4	78.21 (3)	C5—C6—H2c6	109.47
O3—Sr1—O4	115.74 (6)	C5—C6—H2c6^iii^	109.47
Sr1—O1—Sr1^i^	97.34 (4)	C5—C6—H3c6	109.47
Sr1—O2—Sr1^i^	92.08 (5)	C5—C6—H3c6^iii^	109.47
O1—C1—O1^iii^	121.20 (18)	H1c6—C6—H2c6	109.47
O1—C1—C2	119.39 (10)	H1c6—C6—H3c6	109.47
O1^iii^—C1—C2	119.39 (10)	H1c6^iii^—C6—H2c6^iii^	109.47
C1—C2—H1c2	105.88	H1c6^iii^—C6—H3c6^iii^	109.47
C1—C2—H1c2^iii^	105.88	H2c6—C6—H3c6	109.47
C1—C2—C3a	109.54 (19)	H2c6^iii^—C6—H3c6^iii^	109.47
C1—C2—C3a^iii^	109.54 (19)	H1c7—C7—H2c7	109.47
C1—C2—C3b	114.96 (14)	H1c7—C7—H3c7	109.47
C1—C2—C3b^iii^	114.96 (14)	H2c7—C7—H3c7	109.47
H1c2—C2—C3a	110.93	H1c7—H3c7—H2c7	60
H1c2—C2—C3b	105.01	H1ow1—Ow1—H2ow1	103 (4)
H1c2^iii^—C2—C3a^iii^	110.93	H1ow1—Ow1—H3ow1	104 (4)
C2—C3a—H1c3a	109.47	H2ow1—Ow1—H3ow1	104 (5)
C2—C3a—H2c3a	109.47		

Symmetry codes: (i) −*x*+3/2, *y*, −*z*+1/2; (ii) *x*−1/2, *y*, −*z*+1/2; (iii) −*x*+1, *y*, *z*; (iv) *x*−1/2, −*y*+1/2, −*z*; (v) −*x*+3/2, −*y*+1/2, −*z*; (vi) *x*, −*y*, −*z*; (vii) −*x*+1, −*y*, −*z*; (viii) *x*−1/2, *y*−1/2, *z*; (ix) −*x*+2, *y*, *z*; (x) *x*+1/2, −*y*+1/2, −*z*; (xi) *x*+1/2, *y*−1/2, *z*.

### catena-Poly[[µ-aqua-diaqua(µ3-2-methylpropanoato-\ κ4O:O,O':O')strontium] 2-methylpropanoate dihydrate] (II) Hydrogen-bond geometry (Å, º)

**Table:**

*D*—H···*A*	*D*—H	H···*A*	*D*···*A*	*D*—H···*A*
O2—H1*o*2···O5	0.820 (16)	1.936 (15)	2.7465 (14)	169.3 (17)
O3—H1*o*3···O*w*1^xii^	0.827 (17)	2.022 (17)	2.8339 (18)	167.1 (18)
O4—H1*o*4···O5^i^	0.844 (18)	1.964 (17)	2.7887 (16)	165.3 (19)
O*w*1—H1*ow*1···O5^iii^	0.818 (18)	1.976 (19)	2.7913 (18)	174 (2)
O*w*1—H2*ow*1···O*w*1^xiii^	0.82 (2)	1.92 (3)	2.736 (2)	176 (5)
O*w*1—H3*ow*1···O*w*1^xiv^	0.803 (17)	1.946 (17)	2.747 (2)	176 (5)

Symmetry codes: (i) −*x*+3/2, *y*, −*z*+1/2; (iii) −*x*+1, *y*, *z*; (xii) −*x*+1, *y*−1/2, −*z*+1/2; (xiii) −*x*+1/2, *y*, −*z*+1/2; (xiv) −*x*, *y*, *z*.

### catena-Poly[[µ-aqua-diaqua(µ3-2-methylpropanoato-\ κ4O:O,O':O')(calcium/strontium)] 2-methylpropanoate dihydrate] (III) Crystal data

**Table:**

[Ca_0.794_Sr_0.206_(C_4_H_7_O_2_)(H_2_O)_3_]·C_4_H_7_O_2_·2H_2_O	There have been used diffractions with I/σ(I)>20 for the unit cell determination.
*M**_r_* = 314.2	*D*_x_ = 1.358 Mg m^−^^3^
Orthorhombic, *P**b**c**a*	Mo *K*α radiation, λ = 0.71073 Å
Hall symbol: –P 2ac 2ab	Cell parameters from 9952 reflections
*a* = 6.7153 (3) Å	θ = 2.3–27.5°
*b* = 19.6061 (10) Å	µ = 1.08 mm^−^^1^
*c* = 23.3498 (11) Å	*T* = 120 K
*V* = 3074.3 (3) Å^3^	Prism, colourless
*Z* = 8	0.59 × 0.18 × 0.08 mm
*F*(000) = 1342	

### catena-Poly[[µ-aqua-diaqua(µ3-2-methylpropanoato-\ κ4O:O,O':O')(calcium/strontium)] 2-methylpropanoate dihydrate] (III) Data collection

**Table:**

Bruker D8 VENTURE Kappa Duo PHOTON 100 CMOS diffractometer	3533 independent reflections
Radiation source: X-ray tube	2787 reflections with *I* > 3σ(*I*)
Quazar Mo multilayer optic monochromator	*R*_int_ = 0.030
φ and ω scans	θ_max_ = 27.5°, θ_min_ = 2.1°
Absorption correction: multi-scan (*SADABS*; Bruker, 2017)	*h* = −8→8
*T*_min_ = 0.573, *T*_max_ = 0.917	*k* = −25→24
25462 measured reflections	*l* = −30→30

### catena-Poly[[µ-aqua-diaqua(µ3-2-methylpropanoato-\ κ4O:O,O':O')(calcium/strontium)] 2-methylpropanoate dihydrate] (III) Refinement

**Table:**

Refinement on *F*^2^	Primary atom site location: charge flipping
*R*[*F* > 3σ(*F*)] = 0.026	H atoms treated by a mixture of independent and constrained refinement
*wR*(*F*) = 0.062	Weighting scheme based on measured s.u.'s *w* = 1/(σ^2^(*I*) + 0.0004*I*^2^)
*S* = 1.54	(Δ/σ)_max_ = 0.041
3533 reflections	Δρ_max_ = 0.27 e Å^−^^3^
195 parameters	Δρ_min_ = −0.25 e Å^−^^3^
10 restraints	Extinction correction: B-C type 1 Lorentzian isotropic (Becker & Coppens, 1974)
75 constraints	Extinction coefficient: 3100 (900)

### catena-Poly[[µ-aqua-diaqua(µ3-2-methylpropanoato-\ κ4O:O,O':O')(calcium/strontium)] 2-methylpropanoate dihydrate] (III) Fractional atomic coordinates and isotropic or equivalent isotropic displacement parameters (Å^2^)

**Table:**

	*x*	*y*	*z*	*U*_iso_*/*U*_eq_	Occ. (<1)
Ca1	0.51172 (3)	0.147275 (10)	0.285280 (8)	0.01318 (7)	0.7936 (16)
Sr1	0.51172 (3)	0.147275 (10)	0.285280 (8)	0.01318 (7)	0.2064 (16)
O1	0.67331 (12)	0.12224 (5)	0.18768 (4)	0.0183 (3)	
O2	0.34659 (12)	0.11963 (5)	0.18993 (4)	0.0193 (3)	
C1	0.50850 (17)	0.11708 (6)	0.16238 (5)	0.0157 (4)	
C2	0.49970 (18)	0.10867 (7)	0.09788 (6)	0.0197 (4)	
H1c2	0.446005	0.152571	0.082499	0.0236*	
C3	0.3591 (2)	0.05083 (8)	0.08122 (6)	0.0329 (5)	
H1c3	0.350464	0.047978	0.039391	0.0494*	
H2c3	0.410119	0.00765	0.096456	0.0494*	
H3c3	0.22658	0.059708	0.097106	0.0494*	
C4	0.7032 (2)	0.09886 (8)	0.07048 (6)	0.0271 (5)	
H1c4	0.688453	0.096553	0.02877	0.0407*	
H2c4	0.789504	0.137344	0.080535	0.0407*	
H3c4	0.762668	0.056376	0.084481	0.0407*	
O3	0.49864 (13)	0.02977 (5)	0.30914 (5)	0.0278 (3)	
H1o3	0.3934 (13)	0.0071 (7)	0.3132 (7)	0.0417*	
H2o3	0.5919 (16)	0.0012 (6)	0.3097 (7)	0.0417*	
O4	0.76561 (13)	0.23946 (5)	0.24874 (4)	0.0186 (3)	
H1o4	0.734 (2)	0.2655 (6)	0.2217 (4)	0.0279*	
H2o4	0.807 (2)	0.2641 (6)	0.2757 (4)	0.0279*	
O5	0.52603 (13)	0.22321 (5)	0.36693 (4)	0.0223 (3)	
H1o5	0.4290 (15)	0.2502 (6)	0.3690 (7)	0.0335*	
H2o5	0.6251 (14)	0.2493 (6)	0.3680 (6)	0.0335*	
O6	0.69194 (12)	0.30554 (5)	0.14705 (4)	0.0194 (3)	
O7	0.36161 (13)	0.30221 (5)	0.15020 (4)	0.0197 (3)	
C5	0.52316 (17)	0.31043 (6)	0.12361 (5)	0.0151 (3)	
C6	0.51349 (18)	0.33128 (7)	0.06086 (5)	0.0198 (4)	
H1c6	0.381257	0.317376	0.044996	0.0237*	
C7	0.5386 (2)	0.40842 (7)	0.05757 (6)	0.0296 (5)	
H1c7	0.434506	0.430552	0.080392	0.0444*	
H2c7	0.669669	0.421115	0.072612	0.0444*	
H3c7	0.527634	0.423207	0.017606	0.0444*	
C8	0.6670 (2)	0.29505 (8)	0.02419 (6)	0.0333 (5)	
H1c8	0.649839	0.308533	−0.015923	0.05*	
H2c8	0.800974	0.307583	0.037063	0.05*	
H3c8	0.649318	0.245608	0.027732	0.05*	
Ow1	0.22415 (14)	0.42437 (5)	0.19632 (5)	0.0245 (3)	
H1ow1	0.1006 (4)	0.4288 (9)	0.1920 (7)	0.0368*	
H2ow1	0.253 (2)	0.3873 (4)	0.1804 (6)	0.0368*	
Ow2	0.81968 (14)	0.43158 (5)	0.19043 (4)	0.0247 (3)	
H1ow2	0.787 (2)	0.3938 (4)	0.1763 (6)	0.0371*	
H2ow2	0.793 (2)	0.4272 (9)	0.22542 (17)	0.0371*	

### catena-Poly[[µ-aqua-diaqua(µ3-2-methylpropanoato-\ κ4O:O,O':O')(calcium/strontium)] 2-methylpropanoate dihydrate] (III) Atomic displacement parameters (Å^2^)

**Table:**

	*U*^11^	*U*^22^	*U*^33^	*U*^12^	*U*^13^	*U*^23^
Ca1	0.01013 (11)	0.01378 (12)	0.01565 (12)	0.00007 (8)	−0.00019 (8)	−0.00028 (8)
Sr1	0.01013 (11)	0.01378 (12)	0.01565 (12)	0.00007 (8)	−0.00019 (8)	−0.00028 (8)
O1	0.0151 (4)	0.0226 (5)	0.0172 (5)	−0.0002 (4)	−0.0021 (4)	−0.0025 (4)
O2	0.0155 (5)	0.0251 (5)	0.0174 (5)	−0.0001 (4)	0.0011 (4)	−0.0033 (4)
C1	0.0187 (6)	0.0096 (6)	0.0190 (6)	0.0001 (5)	−0.0004 (5)	−0.0004 (5)
C2	0.0224 (7)	0.0211 (7)	0.0155 (6)	−0.0011 (6)	−0.0007 (5)	0.0007 (5)
C3	0.0371 (9)	0.0443 (10)	0.0173 (7)	−0.0170 (8)	−0.0002 (6)	−0.0081 (7)
C4	0.0282 (8)	0.0356 (9)	0.0174 (7)	−0.0058 (7)	0.0041 (6)	−0.0059 (7)
O3	0.0155 (5)	0.0175 (5)	0.0504 (7)	0.0009 (4)	−0.0001 (5)	−0.0016 (5)
O4	0.0240 (5)	0.0167 (5)	0.0151 (5)	−0.0007 (4)	−0.0006 (4)	0.0003 (4)
O5	0.0154 (5)	0.0276 (6)	0.0240 (5)	−0.0002 (4)	0.0007 (4)	0.0028 (4)
O6	0.0168 (4)	0.0239 (5)	0.0176 (5)	0.0003 (4)	−0.0022 (4)	0.0013 (4)
O7	0.0174 (5)	0.0223 (5)	0.0194 (5)	−0.0011 (4)	0.0029 (4)	0.0017 (4)
C5	0.0179 (6)	0.0107 (6)	0.0167 (6)	−0.0006 (5)	0.0000 (5)	−0.0013 (5)
C6	0.0187 (6)	0.0247 (7)	0.0159 (6)	−0.0023 (6)	−0.0016 (5)	0.0012 (5)
C7	0.0421 (9)	0.0257 (8)	0.0210 (7)	0.0039 (7)	−0.0007 (6)	0.0075 (6)
C8	0.0479 (9)	0.0339 (9)	0.0181 (7)	0.0067 (8)	0.0086 (7)	0.0007 (7)
Ow1	0.0189 (5)	0.0206 (6)	0.0341 (6)	−0.0002 (4)	0.0022 (4)	−0.0031 (5)
Ow2	0.0237 (5)	0.0235 (6)	0.0269 (6)	0.0002 (4)	−0.0025 (4)	−0.0035 (5)

### catena-Poly[[µ-aqua-diaqua(µ3-2-methylpropanoato-\ κ4O:O,O':O')(calcium/strontium)] 2-methylpropanoate dihydrate] (III) Geometric parameters (Å, º)

**Table:**

Ca1—Sr1	0	C6—C7	1.524 (2)
Ca1—O3	2.3719 (10)	C6—C8	1.517 (2)
Ca1—O2^i^	2.3845 (9)	C7—H1c7	0.98
Ca1—O1^ii^	2.4091 (8)	C7—H2c7	0.98
Ca1—O5	2.4209 (10)	C7—H3c7	0.98
Ca1—O2	2.5457 (9)	C8—H1c8	0.98
Ca1—O1	2.5714 (9)	C8—H2c8	0.98
Ca1—O4^ii^	2.5747 (9)	C8—H3c8	0.98
Ca1—O4	2.6271 (9)	O3—H1o3	0.840 (11)
Sr1—O1	2.5714 (9)	O3—H2o3	0.840 (12)
Sr1—O1^ii^	2.4091 (8)	O4—H1o4	0.840 (11)
Sr1—O2	2.5457 (9)	O4—H2o4	0.840 (11)
Sr1—O2^i^	2.3845 (9)	O5—H1o5	0.840 (11)
Sr1—O3	2.3719 (10)	O5—H2o5	0.840 (11)
Sr1—O4	2.6271 (9)	Ow1—H1ow1	0.840 (4)
Sr1—O4^ii^	2.5747 (9)	Ow1—H2ow1	0.840 (10)
Sr1—O5	2.4209 (10)	Ow2—H1ow2	0.840 (10)
O1—C1	1.2587 (14)	Ow2—H2ow2	0.840 (5)
O2—C1	1.2644 (15)	C2—C6	4.450 (2)
C1—C2	1.5160 (18)	C2—C8	4.192 (2)
C2—H1c2	1	C2—C8^iii^	4.084 (2)
C2—C3	1.526 (2)	C3—C7^iii^	3.972 (2)
C2—C4	1.5210 (18)	C3—C7^iv^	3.903 (2)
C3—H1c3	0.98	C3—C8^iii^	4.105 (2)
C3—H2c3	0.98	C4—C6^v^	3.9526 (19)
C3—H3c3	0.98	C4—C7^v^	3.746 (2)
C4—H1c4	0.98	C4—C7^vi^	4.128 (2)
C4—H2c4	0.98	C4—C8	4.003 (2)
C4—H3c4	0.98	C4—C8^v^	4.349 (2)
O6—C5	1.2624 (14)	C6—C8^iii^	3.936 (2)
O7—C5	1.2603 (15)	C8—C8^iii^	3.959 (2)
C5—C6	1.5225 (18)	C8—C8^v^	3.959 (2)
C6—H1c6	1		
			
O1—Ca1—O1^ii^	126.26 (3)	Ca1—O2—Ca1^ii^	98.63 (3)
O1—Ca1—O2	50.81 (3)	Ca1—O2—Sr1^ii^	98.63 (3)
O1—Ca1—O2^i^	76.93 (3)	Ca1^ii^—O2—Sr1	98.63 (3)
O1—Ca1—O3	92.20 (3)	Ca1^ii^—O2—O1	160.57 (5)
O1—Ca1—O4	64.51 (3)	Ca1^ii^—O2—O1^ii^	54.25 (2)
O1—Ca1—O4^ii^	97.55 (3)	Ca1^ii^—O2—O3^ii^	51.84 (3)
O1—Ca1—O5	143.00 (3)	Ca1^ii^—O2—O4^ii^	60.18 (3)
O1^ii^—Ca1—O2	76.99 (3)	Ca1^ii^—O2—O5^ii^	48.02 (2)
O1^ii^—Ca1—O2^i^	141.23 (3)	Sr1—O2—Sr1^ii^	98.63 (3)
O1^ii^—Ca1—O3	72.88 (3)	Ca1—O4—Ca1^i^	91.94 (3)
O1^ii^—Ca1—O4	146.87 (3)	Ca1—O4—Sr1^i^	91.94 (3)
O1^ii^—Ca1—O4^ii^	67.56 (3)	Ca1^i^—O4—Sr1	91.94 (3)
O1^ii^—Ca1—O5	87.50 (3)	Sr1—O4—Sr1^i^	91.94 (3)
O2—Ca1—O2^i^	125.08 (3)	O1—C1—O2	120.94 (11)
O2—Ca1—O3	89.00 (3)	O1—C1—C2	120.62 (11)
O2—Ca1—O4	98.35 (3)	O2—C1—C2	118.44 (10)
O2—Ca1—O4^ii^	66.42 (3)	C1—C2—H1c2	106.1
O2—Ca1—O5	146.83 (3)	C1—C2—C3	110.99 (11)
O2^i^—Ca1—O3	75.94 (3)	C1—C2—C4	113.37 (10)
O2^i^—Ca1—O4	67.86 (3)	H1c2—C2—C3	108.97
O2^i^—Ca1—O4^ii^	147.12 (3)	H1c2—C2—C4	106.36
O2^i^—Ca1—O5	84.90 (3)	C3—C2—C4	110.76 (11)
O3—Ca1—O4	140.23 (3)	C2—C3—H1c3	109.47
O3—Ca1—O4^ii^	136.93 (3)	C2—C3—H2c3	109.47
O3—Ca1—O5	114.44 (4)	C2—C3—H3c3	109.47
O4—Ca1—O4^ii^	80.41 (3)	H1c3—C3—H2c3	109.47
O4—Ca1—O5	78.87 (3)	H1c3—C3—H3c3	109.47
O4^ii^—Ca1—O5	80.60 (3)	H2c3—C3—H3c3	109.47
O1—Sr1—O1^ii^	126.26 (3)	C2—C4—H1c4	109.47
O1—Sr1—O2	50.81 (3)	C2—C4—H2c4	109.47
O1—Sr1—O2^i^	76.93 (3)	C2—C4—H3c4	109.47
O1—Sr1—O3	92.20 (3)	H1c4—C4—H2c4	109.47
O1—Sr1—O4	64.51 (3)	H1c4—C4—H3c4	109.47
O1—Sr1—O4^ii^	97.55 (3)	H2c4—C4—H3c4	109.47
O1—Sr1—O5	143.00 (3)	O6—C5—O7	123.33 (11)
O1^ii^—Sr1—O2	76.99 (3)	O6—C5—C6	118.43 (10)
O1^ii^—Sr1—O2^i^	141.23 (3)	O7—C5—C6	118.14 (10)
O1^ii^—Sr1—O3	72.88 (3)	C5—C6—H1c6	108.73
O1^ii^—Sr1—O4	146.87 (3)	C5—C6—C7	108.08 (11)
O1^ii^—Sr1—O4^ii^	67.56 (3)	C5—C6—C8	112.86 (11)
O1^ii^—Sr1—O5	87.50 (3)	H1c6—C6—C7	110.49
O2—Sr1—O2^i^	125.08 (3)	H1c6—C6—C8	105.47
O2—Sr1—O3	89.00 (3)	C7—C6—C8	111.19 (11)
O2—Sr1—O4	98.35 (3)	C6—C7—H1c7	109.47
O2—Sr1—O4^ii^	66.42 (3)	C6—C7—H2c7	109.47
O2—Sr1—O5	146.83 (3)	C6—C7—H3c7	109.47
O2^i^—Sr1—O3	75.94 (3)	H1c7—C7—H2c7	109.47
O2^i^—Sr1—O4	67.86 (3)	H1c7—C7—H3c7	109.47
O2^i^—Sr1—O4^ii^	147.12 (3)	H2c7—C7—H3c7	109.47
O2^i^—Sr1—O5	84.90 (3)	C6—C8—H1c8	109.47
O3—Sr1—O4	140.23 (3)	C6—C8—H2c8	109.47
O3—Sr1—O4^ii^	136.93 (3)	C6—C8—H3c8	109.47
O3—Sr1—O5	114.44 (4)	H1c8—C8—H2c8	109.47
O4—Sr1—O4^ii^	80.41 (3)	H1c8—C8—H3c8	109.47
O4—Sr1—O5	78.87 (3)	H2c8—C8—H3c8	109.47
O4^ii^—Sr1—O5	80.60 (3)	H1o3—O3—H2o3	105.9 (12)
Ca1—O1—Ca1^i^	97.29 (3)	H1o4—O4—H2o4	107.3 (11)
Ca1—O1—Sr1^i^	97.29 (3)	H1o5—O5—H2o5	103.2 (11)
Ca1^i^—O1—Sr1	97.29 (3)	H1ow1—Ow1—H2ow1	105.5 (15)
Ca1^i^—O1—O2	160.27 (5)	H1ow2—Ow2—H2ow2	103.8 (15)
Sr1—O1—Sr1^i^	97.29 (3)		

Symmetry codes: (i) *x*+1/2, *y*, −*z*+1/2; (ii) *x*−1/2, *y*, −*z*+1/2; (iii) *x*−1/2, −*y*+1/2, −*z*; (iv) −*x*+1/2, *y*−1/2, *z*; (v) *x*+1/2, −*y*+1/2, −*z*; (vi) −*x*+3/2, *y*−1/2, *z*.

### catena-Poly[[µ-aqua-diaqua(µ3-2-methylpropanoato-\ κ4O:O,O':O')(calcium/strontium)] 2-methylpropanoate dihydrate] (III) Hydrogen-bond geometry (Å, º)

**Table:**

*D*—H···*A*	*D*—H	H···*A*	*D*···*A*	*D*—H···*A*
O3—H1*o*3···O*w*2^vii^	0.840 (11)	2.061 (12)	2.8767 (14)	163.6 (13)
O3—H2*o*3···O*w*1^vii^	0.840 (12)	1.953 (12)	2.7842 (14)	169.8 (13)
O4—H1*o*4···O6	0.840 (11)	1.932 (10)	2.7498 (13)	164.2 (11)
O4—H2*o*4···O7^i^	0.840 (11)	1.920 (10)	2.7382 (13)	164.2 (11)
O5—H1*o*5···O6^ii^	0.840 (11)	1.964 (11)	2.7831 (13)	164.9 (15)
O5—H2*o*5···O7^i^	0.840 (11)	1.944 (11)	2.7636 (13)	165.0 (14)
O*w*1—H1*ow*1···O*w*2^viii^	0.840 (4)	1.888 (3)	2.7233 (14)	172.8 (16)
O*w*1—H2*ow*1···O7	0.840 (10)	1.951 (11)	2.7836 (14)	170.7 (15)
O*w*2—H1*ow*2···O6	0.840 (10)	1.967 (10)	2.8050 (14)	175.3 (15)
O*w*2—H2*ow*2···O*w*1^i^	0.840 (5)	1.887 (5)	2.7248 (15)	175.2 (17)

Symmetry codes: (i) *x*+1/2, *y*, −*z*+1/2; (ii) *x*−1/2, *y*, −*z*+1/2; (vii) −*x*+1, *y*−1/2, −*z*+1/2; (viii) *x*−1, *y*, *z*.

## References

[bb1] Becker, P. J. & Coppens, P. (1974). *Acta Cryst.* A**30**, 129–147.

[bb2] Brandenburg, K. (2015). *DIAMOND*. Crystal Impact, Bonn, Germany.

[bb3] Bruker (2017). *APEX2*, *SAINT* and *SADABS.* Bruker AXS Inc., Madison, Wisconsin, USA.

[bb4] Coker, E. N., Boyle, T. J., Rodríguez, M. A. & Alam, T. M. (2004). *Polyhedron*, **23**, 1739–1747.

[bb5] Etter, M. C., MacDonald, J. C. & Bernstein, J. (1990). *Acta Cryst.* B**46**, 256–262. 10.1107/s0108768189012929 2344397

[bb6] Gilli, G. & Gilli, P. (2009). *The Nature of the Hydrogen Bond*, p. 61. New York: Oxford University Press Inc.

[bb7] Groom, C. R., Bruno, I. J., Lightfoot, M. P. & Ward, S. C. (2016). *Acta Cryst.* B**72**, 171–179. 10.1107/S2052520616003954 PMC4822653 27048719

[bb8] Itoh, K. (1992). *Ferroelectrics*, **135**, 291–302.

[bb9] Lima-de-Faria, J., Hellner, E., Liebau, F., Makovicky, E. & Parthé, E. (1990). *Acta Cryst.* A**46**, 1–11.

[bb10] Malaestean, I. L., Schmitz, S., Ellern, A. & Kögerler, P. (2013). *Acta Cryst.* C**69**, 1144–1146.

[bb11] Matulková, I., Andreoni, R., Císařová, I., Němec, I. & Fábry, J. (2017). *Z. Kristallogr.* **232**, 471–484.

[bb12] Mishima, N. (1984). *J. Phys. Soc. Jpn*, **53**, 1062–1070.

[bb13] Palatinus, L. & Chapuis, G. (2007). *J. Appl. Cryst.* **40**, 786–790.

[bb14] Petříček, V., Dušek, M. & Palatinus, L. (2014). *Z. Kristallogr.* **229**, 345–352.

[bb15] Scheurell, K., König, R., Troyanov, S. I. & Kemnitz, E. (2012). *Z. Anorg. Allg. Chem.* **638**, 1265–1273.

[bb16] Sheldrick, G. M. (2015). *Acta Cryst.* A**71**, 3–8.

[bb17] Stadnicka, K. & Glazer, A. M. (1980). *Acta Cryst.* B**36**, 2977–2985.

[bb18] Troyanov, S. I., Kiseleva, E. A., Rykov, A. N. & Korenev, Yu. M. (2002). *Zh. Neorg. Khim.* **47**, 1667–1671.

[bb19] Westrip, S. P. (2010). *J. Appl. Cryst.* **43**, 920–925.

